# Diversity of vaginal microbiome and metabolome during genital infections

**DOI:** 10.1038/s41598-019-50410-x

**Published:** 2019-10-01

**Authors:** Camilla Ceccarani, Claudio Foschi, Carola Parolin, Antonietta D’Antuono, Valeria Gaspari, Clarissa Consolandi, Luca Laghi, Tania Camboni, Beatrice Vitali, Marco Severgnini, Antonella Marangoni

**Affiliations:** 1 Institute of Biomedical Technologies, National Research Council, Segrate, Milan Italy; 2 Department of Health Sciences, San Paolo Hospital Medical School, University of Milan, Milan, Italy; 3 Microbiology, Experimental Diagnostic and Specialty Department (DIMES), University of Bologna, Bologna, Italy; 4 Department of Pharmacy and Biotechnology (FaBiT), University of Bologna, Bologna, Italy; 5 Dermatology, St. Orsola-Malpighi Hospital, Bologna, Italy; 6 Centre of Foodomics, Department of Agricultural and Food Sciences, University of Bologna, Cesena, Italy

**Keywords:** Microbiome, Bacterial infection, Fungal infection

## Abstract

We characterized the vaginal ecosystem during common infections of the female genital tract, as vulvovaginal candidiasis (VVC, n = 18) and *Chlamydia trachomatis* infection (CT, n = 20), recruiting healthy (HC, n = 21) and bacterial vaginosis-affected (BV, n = 20) women as references of eubiosis and dysbiosis. The profiles of the vaginal microbiome and metabolome were studied in 79 reproductive-aged women, by means of next generation sequencing and proton based-nuclear magnetic resonance spectroscopy. *Lactobacillus* genus was profoundly depleted in all the genital infections herein considered, and species-level analysis revealed that healthy vaginal microbiome was dominated by *L. crispatus*. In the shift from HC to CT, VVC, and BV, *L. crispatus* was progressively replaced by *L. iners*. CT infection and VVC, as well as BV condition, were mainly characterised by anaerobe genera, e.g. *Gardnerella*, *Prevotella*, *Megasphaera*, *Roseburia* and *Atopobium*. The changes in the bacterial communities occurring during the genital infections resulted in significant alterations in the vaginal metabolites composition, being the decrease of lactate a common marker of all the pathological conditions. In conclusion, according to the taxonomic and metabolomics analysis, we found that each of the four conditions is characterized by a peculiar vaginal microbiome/metabolome fingerprint.

## Introduction

The cervicovaginal ecosystem is made up of diverse microorganisms coexisting in a dynamic balance and establishing complex connections with each other and with the host. In healthy reproductive-aged women, the vaginal microbiome, generally, shows a predominance of *Lactobacillus* genus, and most women display the prevalence of one species among *L. crispatus*, *L. iners*, *L. jensenii* and *L. gasseri*^1^. Lactobacilli promote the maintenance of the vaginal homeostasis and prevent the colonization and growth of adverse microorganisms, including those responsible for sexually transmitted infections (STIs). Such defensive function is exerted through various mechanisms, such as vaginal pH lowering, bioactive compounds production, competition for nutrients and adhesion sites, and modulation of host immune response^2–5^. However, the composition of the vaginal microbiome can vary throughout a woman’s life in response to endogenous and exogenous factors, such as age, pregnancy, pharmaceutical treatments, and urogenital infections.

Urogenital infections and dysbiosis afflict over 1 billion women each year, compromising their wellbeing and affecting their reproductive health^6^. The most common vaginal dysbiosis worldwide is bacterial vaginosis (BV), which is characterized by a shift in the microbial composition from the normally *Lactobacillus*-dominated to a high-complexity, polymicrobial community. It has been largely established that BV condition is characterized by the presence of anaerobic bacteria, such as *Gardnerella vaginalis*, *Atopobium* spp., *Prevotella* spp., and high concentrations of various biogenic amines (putrescine, cadaverine and trimethylamine), short chain fatty acids (especially acetate and succinate) and low levels of some amino acids (tyrosine, glutamate)^7–9^. In several epidemiological studies it has been reported that BV represents a risk factor for the acquisition of STIs^10,11^.

Among infections, vulvovaginal candidiasis (VVC) affects about 75% of reproductive-aged women at least once during their lives, with about 5% of them suffering of recurrences^12^. VVC is caused by *Candida* spp. which, in particular conditions, instead of being part of the normal vaginal microflora, become a robust opportunistic fungal pathogen, with a tendency to overgrow^13^. *C. albicans* is responsible for 80–92% of VVC cases. The causes and phases of the transition from the homeostasis to the pathogenic state of VVC are not clear yet, as well as the impact, if any, on the vaginal microbiome structure and metabolomic profile.

*Chlamydia trachomatis* (CT) represents the most common bacterial STI worldwide, and new infections probably exceed 131 million per year^14^. CT is a Gram-negative, obligate intracellular bacterial pathogen with a unique biphasic developmental cycle^15^. CT serovars from D to K are responsible for common and frequently asymptomatic urogenital infections (i.e. urethritis and cervicitis), that may lead to several sequelae and complications, including pelvic inflammatory disease (PID), ectopic pregnancy, and infertility. Globally, serovars E, F, D, and G represent the most common serovars, accounting for 60–80% of cases^16,17^. Given its high prevalence and sociological impact, correlation of CT infection with the cervico-vaginal microbiome has recently gained particular attention: some cross-sectional studies have been published^18,19^, and vaginal microbiome/metabolome fingerprints have been explored^20^.

In this work, we depicted and compared the alterations that occur in the vaginal microbiome and metabolome during common genital infections. Two high-performing techniques, i.e. next generation sequencing (NGS) and proton-based nuclear magnetic resonance (^1^H-NMR) spectroscopy, were applied to unravel the vaginal microbiome composition and the metabolome fingerprint, respectively, highlighting common trends and correlations. The relatively-less characterized ecosystems corresponding to VVC and CT infection were compared to the healthy status (reference of eubiosis) and BV condition (reference of dysbiosis).

## Results

### Study group

A total of 79 patients were included in the study: specifically, 21 were considered healthy (HC), 20 received a diagnosis of bacterial vaginosis (BV), 20 had a *C. trachomatis* genital infection (CT) and 18 suffered from vulvo-vaginal candidiasis (VVC) due to *C. albicans*. Clinical and demographic information of the study groups are reported in details in Table 1. No significant differences were found in the age distribution between the different groups of women. Most of the HC and CT-positive women showed a Nugent score 0–3 (76% and 65%, respectively), while 60% of BV subjects were characterised by a Nugent score >7. Women in the VVC group had an intermediate condition (Nugent score 4–6) in 61% of cases. A creamy gray discharge was referred by 85% of BV patients, whereas all subjects suffering from VVC complained from itching and/or whitish discharge. More than half (11/20; 55%) of CT-positive patients were completely asymptomatic. The most common CT serovar in our population was E (11/20; 55%), followed by F (4/20; 20%), G (2/20; 10%), D (2/20; 10%) and K (1/20; 5%).

**Table 1:** Demographic and clinical characteristics of the women enrolled for the study, subdivided in healthy (HC), bacterial vaginosis (BV), *C. trachomatis* (CT), and vulvo-vaginal candidiasis (VVC) groups.

	HC (N = 21)	BV (N = 20)	CT (N = 20)	VVC (N = 18)	*P*
Enrolment criteria	No symptoms and negative for microbiological tests	Positive for 3/4 Amsel criteria and Nugent score > 3	Vaginal detection of CT DNA by NAAT	Suggestive symptoms and detection of *C. albicans*	—
Mean age ± SD (years)	26.1 ± 6.3	29.3 ± 7.7	24.3 ± 3.4	27.8 ± 7.2	0.08
Mean BMI ± SD (kg/m^2^)	23.8 ± 2.4	22.9 ± 2.4	23.6 ± 1.8	23.0 ± 2.0	0.38
**Amsel criteria**					
Creamy gray discharge	0/21 (0.0%)	17/20 (85.0%)	0/20 (0.0%)	0/18 (0.0%)	<0.0001
Mean vaginal pH value	4.1 ± 0.3	4.8 ± 0.4	4.2 ± 0.4	4.3 ± 0.5	<0.0001
Positive Whiff test^a^	5/21 (23.8%)	10/20 (50.0%)	4/20 (20.0%)	3/18 (16.7%)	0.07
Clue cells present	1/21 (4.7%)	19/20 (95.0%)	3/20 (15.0%)	5/18 (27.8%)	<0.0001
**Nugent score**					
0–3	16/21 (76.2%)	0/20 (0.0%)	13/20 (65.0%)	7/18 (38.9%)	<0.0001
4–6	5/21 (23.8%)	8/20 (40.0%)	7/20 (35.0%)	11/18 (61.1%)	0.11
7–10	0/21 (0.0%)	12/20 (60.0%)	0/20 (0.0%)	0/18 (0.0%)	<0.001

^a^The Whiff test is considered positive when, by adding a drop of 10% KOH to a microscopic slide containing the vaginal secretions, a characteristic ‘fishy’ odour is present.

### Vaginal microbiota structure characterization

**Figure 1:**
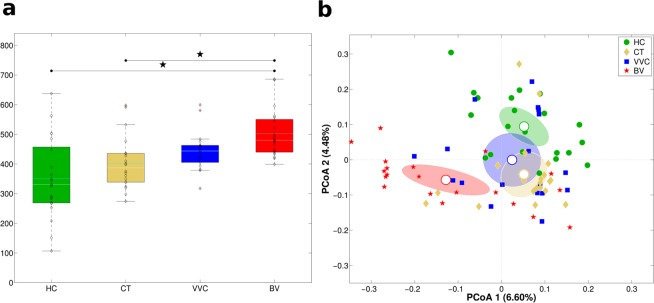
Structure of the vaginal microbiota. Microbial composition of vaginal swabs of healthy (HC), *C. trachomatis* (CT), vulvo-vaginal candidiasis (VVC) and bacterial vaginosis (BV) positive women was analysed. (**a**) Boxplots of Shannon index (α-diversity). (**b**) Principal Coordinates Analysis (PCoA) plot based on unweighted Unifrac distance (β-diversity). Each point corresponds to a sample. For each experimental class, the SEM-based confidence ellipse around the centroid is depicted. The first two components of the variance are represented.

DNA extracted from vaginal swabs was subjected to next generation sequencing of the V3-V4 hypervariable regions of 16S rRNA gene, which produced an average of 56,273 reads per sample. Alpha-diversity analysis using Shannon index metrics showed significant differences in biodiversity among the four groups (Kruskal-Wallis p-value < 0.001, Fig. 1a). In particular, BV samples were characterised by a significant higher microbiota diversity compared to HC (p = 0.006) and CT (p = 0.006) groups; the comparison between HC and BV resulted significant with the Chao1 index as well (p = 0.024). No significant differences were observed with the other metrics used (observed species and Faith’s phylogenetic diversity index).

Beta-diversity analysis highlighted that each condition was characterized by a peculiar microbiota: principal coordinates analysis (PCoA) showed a significant segregation of the four women groups (adonis p-value < 0.001, on both weighted and unweighted Unifrac distances, Fig. 1b). Moreover, a significant separation on unweighted Unifrac distance was evidenced for all pairwise comparisons (all p-values < 0.05), being BV the most profoundly different condition compared to the others. Weighted Unifrac distances analysis confirmed that HC and BV possessed a unique microbial composition (all pairwise comparison p-values < 0.05), whereas VVC and CT conditions, although different from the other two experimental groups, did not show a significant separation between them (p = 0.34). Moreover, within the CT-infected group, no significances were observed, in both alpha and beta diversities, according to *Chlamydia* serotype (serovar E: 11 patients; non-E, including serovars F, G, D, K: 9 patients).

### Taxonomic composition of vaginal bacterial communities

**Figure 2:**
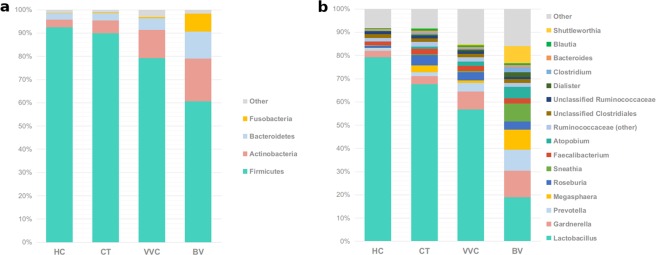
Taxonomic composition of the vaginal microbiota. Stacked bar charts of taxonomy relative abundances at (**a**) phylum and (**b**) genus level for healthy (HC), *C. trachomatis* (CT), vulvo-vaginal candidiasis (VVC) and bacterial vaginosis (BV) positive subjects. Only phyla and genera present at relative abundances >1% in at least 20% (i.e.: ≥16 samples) are reported. Remaining taxa are grouped in the “Other” category.

Analyzing taxonomic classification at the phylum level, it was found that all samples were mainly constituted by Firmicutes, Actinobacteria, and Bacteroidetes, although with many significant differences in their abundances across groups (all Kruskal-Wallis p-values < 0.001, Fig. 2a). Firmicutes dominated the vaginal microbiota of all women groups, with abundance ranging from 92.4% in HC, to 60.6% in BV-affected women. Besides BV, also VVC-infected women (79.2%), but not CT subjects (89.8%) were significantly depleted in Firmicutes. In contrast, Actinobacteria were significantly more abundant in BV (18.4%), and VVC (12.0%) groups with respect to HC (3.4%) and CT (5.6%). A similar observation was made for Bacteroidetes phylum, which represents 11.5% of abundance in BV women, 5.0% in VVC and only 2.7% and 3.0% in HC and CT subjects, respectively. Fusobacteria phylum was significantly enriched only in BV samples (7.9%), while Proteobacteria mainly characterized the VVC group (2.2%).

At a lower taxonomic level, *Lactobacillaceae* family (Kruskal-Wallis p-value < 0.001), almost totally represented by *Lactobacillus* genus, was found predominant in healthy women (79.2%), as well as in CT (67.5%) and VVC (56.7%) subjects, but profoundly and significantly depleted in BV subjects (18.8%). *Lachnospiraceae*, *Bifidobacteriaceae*, and *Veillonellaceae* families were differently represented across groups (Kruskal-Wallis p-values: p < 0.005 for all families, Table 2); in particular, they were significantly more abundant in all the pathological conditions with respect to healthy women.

**Table 2:** Average relative abundances of main microbial groups (phylum, family, genus).

Level		Group	Relative abundance				P-value			
			HC (%)	CT (%)	VVC (%)	BV (%)	Overall^a^	CT *vs*. HC^b^	VVC *vs*. HC^b^	BV *vs*. HC^b^
Phylum	↓	Firmicutes	92.41	89.78	79.22	60.61	<0.001	—	0.004	<0.001
	↑	Actinobacteria	3.39	5.62	12.02	18.38	<0.001	—	<0.001	<0.001
	↑	Bacteroidetes	2.71	2.99	5.02	11.51	<0.001	—	0.019	<0.001
	↑	Fusobacteria	0.22	0.42	0.54	7.87	0.003	—	—	<0.001
	↑	Proteobacteria	0.57	0.60	2.23	0.41	0.030	—	0.013	—
Family	↓	*Lactobacillaceae*	79.19	67.50	56.75	18.83	<0.001	—	0.004	<0.001
	↑	*Lachnospiraceae*	3.16	8.10	6.61	13.67	0.003	<0.001	0.007	0.007
	↑	*Bifidobacteriaceae*	2.93	4.17	9.56	11.82	<0.001	0.044	<0.001	<0.001
	=	*Ruminococcaceae*	5.22	6.59	6.29	6.21	—	—	—	—
	↑	*Veillonellaceae*	0.74	3.72	2.42	10.91	<0.001	0.031	0.004	<0.001
	↑	*Prevotellaceae*	1.16	1.60	3.76	9.15	<0.001	—	0.002	<0.001
	↑	*Coriobacteriaceae*	0.32	1.30	2.29	5.96	<0.001	0.031	<0.001	<0.001
	↑	*Leptotrichiaceae*	0.20	0.41	0.53	7.77	0.002	—	—	<0.001
	=	Uncl. *Clostridiales*	1.66	1.56	1.44	1.58	—	—	—	—
	↑	*Tissierellaceae*	1.14	0.29	1.23	3.04	<0.001	—	0.044	<0.001
	↑	*Aerococcaceae*	0.03	0.24	1.51	2.07	<0.001	0.034	—	<0.001
	↑	*Clostridiaceae*	0.19	0.69	0.36	2.17	<0.001	0.009	—	<0.001
	=	*Bacteroidaceae*	0.69	0.91	0.81	0.86	—	—	—	—
	=	*Streptococcaceae*	0.42	0.50	1.05	0.55	—	—	—	—
	=	*Pasteurellaceae*	0.03	0.10	1.46	0.02	—	—	—	—
	=	*Porphyromonadaceae*	0.38	0.13	0.09	0.83	—	—	—	—
	↑	*Gemellaceae*	0.06	0.06	0.82	0.28	0.012	—	0.013	0.005
	↑	*Actinomycetaceae*	0.06	0.09	0.10	0.53	0.010	—	0.029	0.006
	↑	*Peptostreptococcaceae*	0.04	0.01	0.08	0.64	0.011	—	0.017	0.002
Genus	↓	*Lactobacillus*	79.16	67.45	56.69	18.80	<0.001	—	0.004	<0.001
	↑	*Gardnerella*	2.72	3.65	7.68	11.44	<0.001	—	0.003	<0.001
	↑	*Prevotella*	1.16	1.60	3.76	9.15	<0.001	—	0.002	<0.001
	↑	*Megasphaera*	0.06	2.97	1.04	8.64	<0.001	0.001	0.036	<0.001
	↑	*Roseburia*	1.09	4.42	3.51	3.51	<0.001	<0.001	<0.001	0.031
	↑	*Sneathia*	0.18	0.41	0.53	7.76	<0.001	0.020	—	<0.001
	=	*Shuttleworthia*	0.03	0.54	0.31	7.48	—	—	—	—
	↑	*Faecalibacterium*	1.49	2.31	2.14	2.09	0.027	0.003	0.041	—
	↑	*Atopobium*	0.17	1.00	1.94	4.92	<0.001	0.024	0.001	<0.001
	↑	*Ruminococcaceae* (other)	1.35	1.95	1.86	1.81	0.023	0.003	0.041	—
	=	Uncl. *Clostridiales*	1.66	1.56	1.44	1.58	—	—	—	—
	=	Uncl. *Ruminococcaceae*	1.08	1.03	1.02	1.03	—	—	—	—
	↑	*Aerococcus*	0.03	0.24	1.50	2.06	<0.001	0.021	—	<0.001
	↑	*Dialister*	0.37	0.56	0.78	2.02	<0.001	0.011	<0.001	<0.001
	↑	*Clostridium*	0.16	0.65	0.32	2.14	<0.001	0.008	—	<0.001
	↑	*Bacteroides*	0.69	0.91	0.81	0.86	—	—	—	—
	↑	*Blautia*	0.35	0.91	0.77	0.74	<0.001	<0.001	0.004	—
	=	*Coprococcus*	0.61	0.66	0.63	0.59	—	—	—	—
	↑	*Streptococcus*	0.40	0.49	1.04	0.54	0.045	0.012	0.013	—
	=	*Oscillospira*	0.65	0.56	0.53	0.58	—	—	—	—
	↑	*Ruminococcus*	0.54	0.58	0.59	0.56	—	—	—	—
	↑	*Bifidobacterium*	0.20	0.46	1.28	0.33	<0.001	<0.001	0.005	—
	↑	Uncl. *Lachnospiraceae*	0.40	0.59	0.54	0.52	0.029	0.006	—	—
	=	*Parvimonas*	0.20	0.07	0.17	1.39	<0.001	—	—	<0.001
	=	*Peptoniphilus*	0.35	0.15	0.13	1.09	—	—	—	—
	=	*Haemophilus*	0.02	0.10	1.42	0.02	—	—	—	—
	↑	Uncl. *Coriobacteriaceae*	0.10	0.19	0.24	0.89	<0.001	0.023	0.017	<0.001
	=	*Akkermansia*	0.30	0.39	0.35	0.32	—	—	—	—
	=	*Anaerococcus*	0.48	0.03	0.46	0.39	—	—	—	—
	↑	*Escherichia*	0.13	0.33	0.40	0.23	<0.001	<0.001	<0.001	—
	=	*Porphyromonas*	0.25	0.05	0.01	0.73	—	—	—	—
	↑	*Ureaplasma*	0.25	0.04	0.41	0.09	<0.001	0.001	—	0.005
Genus	↑	*Peptostreptococcus*	0.03	<0.01	0.08	0.63	0.018	—	0.024	0.009
	↑	*Finegoldia*	0.09	0.03	0.45	0.16	0.016	—	0.015	—
	↑	*Veillonella*	0.08	0.07	0.46	0.08	0.038	—	0.019	—
	↑	*Gemella*	0.05	0.03	0.32	0.27	<0.001	—	—	<0.001
	↑	*Mobiluncus*	0.03	0.08	0.02	0.49	0.013	—	—	0.002
	↑	*Alloscardovia*	<0.01	0.06	0.57	<0.01	0.002	0.041	<0.001	—
	↑	Uncl. *Gemellaceae*	0.01	0.03	0.49	<0.01	0.019	—	—	0.041
	=	Uncl. Rs-045	<0.01	<0.01	0.04	0.43	—	—	—	—

For each taxa, significant p-values of the non-parametric Kruskal-Wallis test and of pairwise comparison (Dunn’s test) of women affected by *Chlamydia trachomatis* infection (CT), vulvo-vaginal candidiasis (VVC) or bacterial vaginosis (BV) versus healthy women (HC) are reported. Arrows indicate the direction of the variation with respect to HC: ↓, decreased; ↑, increased; =, unchanged.

^a^P-value of the Kruskal-wallis test for equal medians among groups.

^b^P-value of the Dunn’s post-hoc test test for equal medians between each pathological condition (i.e.: CT, VVC or BV) and HC.

Analysing the vaginal microbiota composition at the genus level, the aforementioned progressive depletion in *Lactobacillus* genus from HC, to CT, VVC, and BV conditions was associated to a corresponding increase in the anaerobe genera *Gardnerella*, *Prevotella*, *Megasphaera*, *Roseburia* and *Atopobium* (all Kruskal-Wallis p-values < 0.001, Fig. 2b). With respect to HC vaginal microbiota composition, *Gardnerella* and *Prevotella* were found significantly increased in BV and VVC women only, whereas *Megasphaera*, *Roseburia* and *Atopobium* were significantly increased in CT subjects too. Moreover, *Faecalibacterium* was significantly increased in both CT and VVC, but not in BV. Finally, BV vaginal microbiota also showed a significant increase in *Sneathia* (Table 2). Notably, 4/20 women affected by BV showed the presence of *Shuttleworthia* at very high relative abundances (range: 29.1–41.5%), compared to an average of 0.23% in other samples. Among other bacteria, *Mycoplasma* was detected at very low relative abundance (0.07%) and resulted increased (p < 0.05) in BV with respect to HC and CT; *Chlamydia*, on the other hand, was detected only in CT subjects, as expected, although at a very low relative abundance (0.02%) (data not shown).

### Species-level analysis

**Figure 3:**
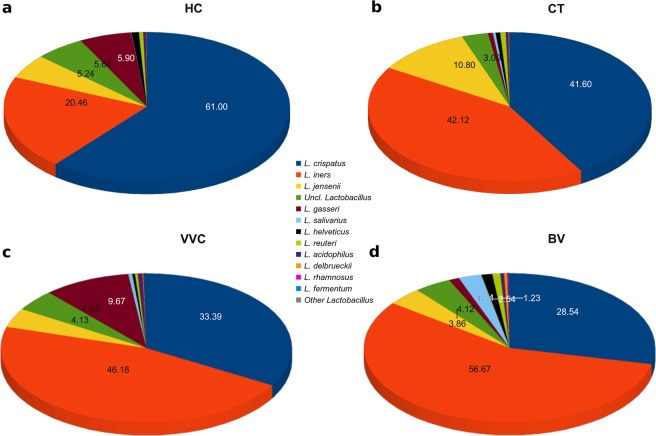
Species-level characterization of *Lactobacillus* genus. Sequences classified within *Lactobacillus* genus were characterized down to species level by classifying them against a custom reference database. Proportions are relative to the total amount of *Lactobacillus* genus in each group. Only the twelve most-abundant species are represented. Less abundant *Lactobacillus* species are grouped under “Other *Lactobacillus*”. Per-sample relative abundances were averaged in each experimental category: (**a**) healthy (HC), (**b**) *C. trachomatis* (CT), (**c**) vulvo-vaginal candidiasis (VVC) and (**d**) bacterial vaginosis (BV) positive women.

Because of the crucial importance of *Lactobacillus* species in the vaginal environment, a focus on this genus has been performed (Fig. 3). The *Lactobacillus* species-level analysis revealed that the vaginal microbiota of healthy subjects was dominated by *L. crispatus* for up to 61.0% of the total *Lactobacillus* sequences, while the shift towards a pathological condition was associated to a significant reduction in this species abundance (p = 0.011), which represented 41.6% in CT infected women, 33.4% in VVC (p = 0.006 *vs* HC) and 28.5% in BV subjects (p = 0.005 *vs* HC). Such a depletion was reflected in a corresponding increase of *L. iners* in the shift from the healthy status (20.5%) to CT infection (42.1%), VVC (46.2%, p = 0.01 *vs* HC), and BV (56.7%, p = 0.009 *vs* HC). Interestingly, *L. gasseri* mainly characterized the VVC group (9.7%, p = 0.005 *vs* HC), whereas BV subjects showed the highest diversity of *Lactobacillus* species, including *L. salivarius, L. helveticus, L. delbrueckii*, and *L. rhamnosus*.

Besides the *Lactobacillus* species, the SPINGO-based bioinformatic analysis allowed to further refine the taxonomic classification of several bacterial genera found as differentially abundant in HC *vs* CT, VVC or BV comparisons. Identified species were *Gardnerella vaginalis*, *Faecalibacterium prausnitzii*, *Megasphaera elsdenii*, *Atopobium vaginae*, *Shuttleworthia satelles* and *Chlamydia trachomatis*. *Sneathia* genus comprised both *S. amnionii* and *S. sanguinegens*. Within *Dialister* genus, we detected *D. succinatiphilus*, plus other unidentified members; *Prevotella* species included, among others, *P. bivia*, *P. amnii*, and *P. timonensis*; the majority (>97%) of *Mycoplasma* sequences were identified as *M. hominis*. Finally, no species-level characterization was obtained for *Roseburia*.

### Taxonomic co-abundance clusters

To identify patterns of co-expression in vaginal microbiota, we established co-abundant genera associations on the whole dataset and clustered them into five co-abundance groups (CAGs), whose names were assigned according to the most representative genera. Three groups resulted composed by only one genus each, one of them being *Lactobacillus*, the major player in vaginal microbiota, the other two (i.e.: *Clostridium* and *Finegoldia*) being sub-dominant genera. Another group, named as *Roseburia* CAG, comprised several gut-specific commensal genera (e.g.: *Faecalibacterium*, *Ruminococcus*, *Akkermansia*), summing up to 14.2% of total relative abundance. The last group, namely *Gardnerella* CAG, included opportunistic genera (e.g.: *Gardnerella* itself, *Atopobium*, *Megasphaera*), averaging 19.3% of the total relative abundance (Suppl. Fig. 1).

In HC, *Lactobacillus* accounted for the vast majority of the relative abundance and was negatively correlated with all the other CAGs. In CT-affected women, on the other hand, an increase in *Clostridium*, *Gardnerella* and *Roseburia* CAGs, positively correlated one to each other, was observed. Moreover, in VVC, we detected an increase of *Finegoldia* and *Gardnerella* CAG, the latter showing a mixed (partly positive and partly negative) correlation to *Roseburia* CAG. Finally, in BV women, there was a substantial increase in *Gardnerella* CAG (which reflected that of many of its members), together with an increase of *Clostridium* and of the proportion of bacteria not belonging to any of the identified CAGs; notably, *Roseburia* and *Gardnerella* CAGs were negatively correlated, whereas *Lactobacillus* showed a moderate positive correlation to *Roseburia* CAG, indicating a consistent switch in the relationship between bacterial groups (Suppl. Fig. 2).

### Vaginal metabolites composition for VVC-affected women and metabolite-microbiota correlation

**Figure 4:**
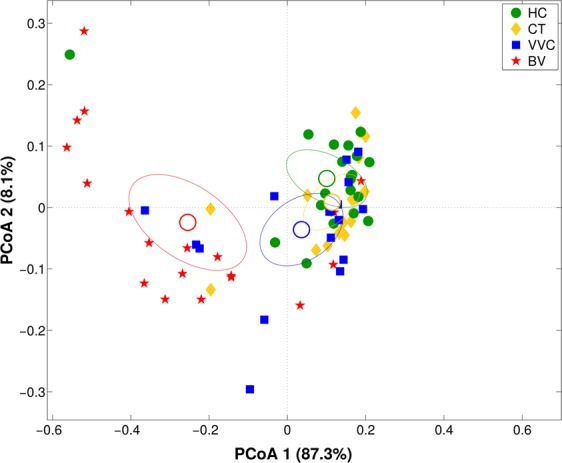
Vaginal metabolome analysis. Principal Coordinates Analysis (PCoA) on Bray-Curtis distance showing the separation of the samples according to metabolite profile as obtained from ^1^H-NMR analysis of vaginal samples from healthy (HC), *C. trachomatis* (CT), vulvo-vaginal candidiasis (VVC) and bacterial vaginosis (BV) positive women. The first and second components of the total variance are shown.

In this paper, we analyzed the metabolites composition of VVC samples by ^1^H-NMR, and compared it to the metabolomics data already obtained for the other groups^20^. Multiple comparison of metabolic profiles revealed a peculiar fingerprint of VVC-affected women, which resulted significantly separated from HC (p < 0.02 on both Euclidean and Bray-Curtis distances) and from BV (p < 0.002 on both distances). No significant differences were found between VVC and CT groups (Fig. 4).

**Figure 5:**
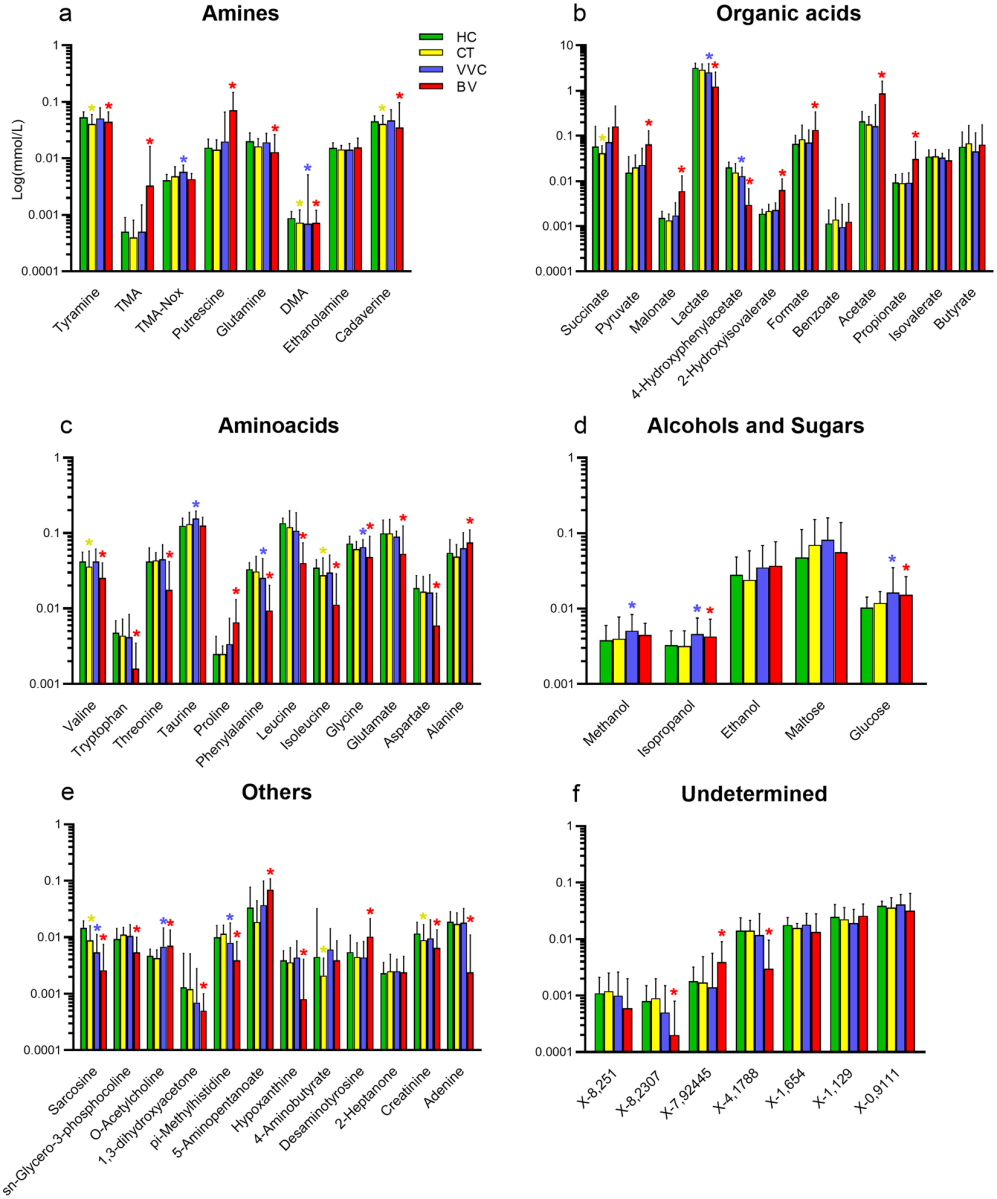
Metabolite concentration in vaginal samples. Bar plots showing metabolites concentrations per vaginal samples from healthy (HC), *C. trachomatis* (CT), vulvo-vaginal candidiasis (VVC) and bacterial vaginosis (BV) positive women. Each bar represents the median metabolite concentration (mmol/L) across replicates per experimental group. Error bars show inter-quartile range (i.e.: 75^th^–25^th^ quantile). Metabolites are grouped according to biochemical type (panels a–f). Stars indicate significant differences *vs* HC group.

When compared to the healthy group, VVC metabolic profile resulted enriched (p < 0.05) in TMA-Nox (TMAO), taurine, and methanol, and, similarly to BV samples, in isopropanol, O-acetylcholine and glucose. In addition, significantly lower concentrations (p < 0.05) of lactate, 4-hydroxyphenylacetate, phenylalanine, pi-methylhistidine and glycine were detected in VVC and BV women; dimethylamine (DMA) and sarcosine were found significantly diminished in all pathological conditions compared to healthy subjects (Fig. 5).

**Figure 6:**
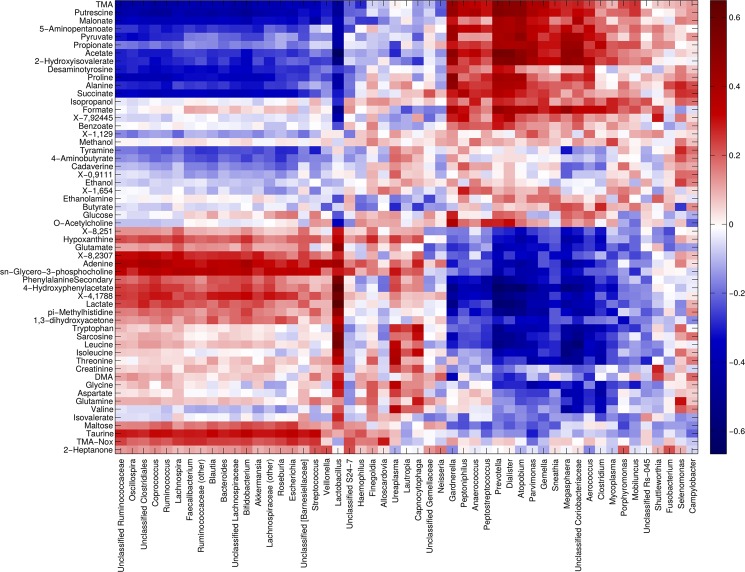
Correlation between metabolome and microbiota. Heatmap showing the Spearman’s correlation coefficient between metabolites concentration and the relative abundances of the main bacterial genera. Only groups present at >1% of relative abundance in at least one sample were considered. Metabolite and microbial data were clustered using Pearson’s correlation metric and average linkage.

*Lactobacillus* abundance was strongly positively associated with lactate and 4-hydroxyphenylacetate concentrations (Spearman rank coefficient, ρ: 0.65 for both) and, to a lesser extent, to the concentration of several amino acids, such as isoleucine, leucine, tryptophan, phenylalanine and aspartate (ρ ranging from 0.28 to 0.58), DMA (ρ = 0.28), sarcosine (ρ = 0.54) and pi-methylhistidine (0.49). On the other hand, *Lactobacillus* abundance was negatively correlated to formate (ρ = −0.51), acetate (ρ = −0.67), 2-hydroxyisovalerate (ρ = −0.55) and alanine (ρ = −0.43) concentrations (Fig. 6). Other bacterial genera (i.e.: *Gardnerella, Prevotella, Megasphaera, Atopobium, Dialister* and *Clostridium*) showed a negative correlation with the aforementioned metabolites positively related to *Lactobacillus*, with coefficients ranging from −0.24 to −0.64, while they showed a positive correlation to organic acids (i.e.: formate, pyruvate, propionate, acetate, 2-hydroxyisovalerate), amines (i.e.: TMA, putrescine), amino acids (i.e.: proline and alanine) and 5-aminopentanoate (ρ = 0.27–0.57). Finally, a third group of bacteria, including *Roseburia*, *Blautia*, *Streptococcus* and *Bifidobacterium*, was moderately positively correlated to TMAO and taurine (ρ = 0.24–0.33). All of these correlation coefficients were statistically significant (*p* < 0.05). Bacterial and metabolite-derived data were characterized by a very high and significant association, as demonstrated by Procrustes analysis (permutation-based p-value of m^2^ goodness-of-fit estimation <0.001) and RV coefficient (0.89, p < 0.001, for all phylogenetic levels).

## Discussion

In this work, we investigated the modifications that take place in the vaginal microbiome and metabolome during common genital infections, namely VVC and CT infection, in comparison with the thoroughly studied conditions corresponding to health and BV. Data generated by high-performing techniques, i.e. NGS and ^1^H-NMR spectroscopy, depicted in details the composition of the vaginal microbiome and metabolome and, notably, their correlations, in order to identify hallmarks of these common genital infections.

To the best of our knowledge, this paper analyzes in depth, for the first time, the vaginal microbiome and metabolome of VVC affected women. VVC represents the most frequent mucocutaneous mycosis caused by yeasts of the genus *Candida*, with a significant impact on women’s quality of life and associated medical costs, because of the high rate of recurrences and the increasing level of antifungal resistance^21^. In this context, a deep knowledge about the vaginal ecosystem during *Candida* infections could contribute to better understand VVC pathogenesis and to set up new strategies for its control.

According to the taxonomic and metabolomics analysis, we have found that each of the four conditions herein considered is characterized by a peculiar fingerprint.

Vaginal microbiota of HC was dominated, as expected, by members of the *Lactobacillus* genus and, in particular, by *L. crispatus* species. *Lactobacillus* showed a trend towards a decrease in CT women, associated to a significant increase in *Megasphaera*, *Atopobium*, *Faecalibacterium* and *Roseburia*. The increase of *Faecalibacterium prausnitzii* in vaginal flora has already been recognized as a risk factor for *C. trachomatis* infection acquisition^22^. Similarly, Balle and colleagues found that some taxa were differentially abundant in the cervico-vaginal microbiota between *C*. *trachomatis*-infected and uninfected women, including *G*. *vaginalis*, *A*. *vaginae*, *Dialister* spp., *Prevotella* spp. and *Megasphaera* (all BV-associated bacteria)^23^.

On the other hand, microbiota of BV-affected women was characterized by a major shift in the relative abundance of many groups, such as *Lactobacillus* (decreased), *Gardnerella*, *Prevotella*, *Megasphaera*, *Roseburia*, *Sneathia*, and *Atopobium* (all increased). Increase of *Mobiluncus* was also observed, even if at a low relative abundance (0.49% and 0.03% in BV and HC, respectively, p = 0.002), confirming evidences from Parolin *et al*.^20^. BV condition also presented a peculiar network of co-abundance among bacterial groups, as *Lactobacillus* was positively correlated to *Roseburia* CAG, which is, in turn, negatively correlated to *Gardnerella* group.

VVC microbiota profile was found as significantly altered, too, bearing many features in common with BV: all bacterial genera altered in BV, with the exception of *Sneathia* and with the addition of *Faecalibacterium*, showed a significant shift also in VVC, suggesting that *Candida* spp. infections reflect into a considerable modification of the vaginal ecology. Notably, *Faecalibacterium* and *Roseburia*, two major players in the healthy gut ecosystem, are associated in the vaginal environment to a proliferation of opportunistic microorganisms and to a general dysbiosis. It should be remembered that the gut could be the initial source of the vaginal colonisation by *Candida* spp., with the creation of a persistent intestinal reservoir of the yeast^24^. Thus, the presence of *Faecalibacterium* and *Roseburia* in the vaginal niche could reflect a general translocation of various microorganisms from the gastrointestinal tract to the vagina.

The trend towards the reduction of the relative abundance of *L. crispatus* and the corresponding increase of *L. iners* was found as another fingerprint of the ongoing dysbiosis in infectious conditions. *L. crispatus* has been reported to reduce the adherence of *G. vaginalis* and *C. trachomatis* to epithelial cells^25^, while *L. iners* has not^26^. A potential explanation lies in the fact that *L. iners* does not produce D-lactic acid, as well as H_2_O_2_, suggesting a lower protective ability against pathogens as compared to other *Lactobacillus* species^27^. Indeed, a *L. iners*-dominated community was associated with an increased risk for the acquisition of *C. trachomatis* genital infections^22^. Similarly, an increase of non-H_2_O_2_-producing *L. iners*, with a contemporary decrease in H_2_O_2_-producing *Lactobacillus* species, has been observed in VVC-positive women^28^. Thus *L. iners* is considered to be a transitional species, colonizing after perturbations of the vaginal environment, whereas a *L. crispatus*-predominant microbiota represents the hallmark of a healthy and stable vaginal status^29,30^.

Metabolome analysis by ^1^H-NMR spectroscopy provided a further explanation of the ongoing changes in genital infections as compared to HC. The production of lactic acid by *Lactobacillus* species, *L. crispatus* in particular (connected to the concentration of its conjugate base lactate), acidifies the pH in the vaginal environment, contributing in keeping the environment in a homeostasis against growth of potentially harming microorganisms^31^. Lactic acid bacteria species are known producers of branched-chain amino acids^32^, thus the higher concentration of some of them, such as valine, leucine, and isoleucine, is another hallmark of the prevalence of lactobacilli in HC women.

On the other hand, the sharp decrease in lactate concentration in CT, VVC and BV, with the consequent increase of pH, is a marker of dysbiosis: the proliferation of diverse bacterial genera, some of them typical of the gut/fecal microbiota^31^, is further associated to the increased presence of short-chain fatty acids, such as butyrate, propionate and acetate.

Moreover, all three infection conditions are characterized by a significant reduction of DMA concentration, with a corresponding trend towards the increase of TMA (which reaches a significant level in BV), suggesting that vaginal dysbiosis impaired the balance between these two molecules belonging to the same pathway of choline degradation^33^.

In accordance with previous investigations^7,20^, we found that BV women are characterized by higher levels of biogenic amines and short-chain organic acids. Moreover, we reinforced the hypothesis of the role of *Mycoplasma hominis* in BV condition^34^, highlighted by both its increased relative abundance and the positive correlation between *Mycoplasma* and BV-related metabolites (e.g. formate, putrescine, propionate, acetate).

The vaginal metabolic profiles of CT-infected women show only slight modifications compared to healthy controls, being the reduction in some amino acids and biogenic amines the most significant variations. Presence of *C. trachomatis* was assessed only in CT subjects, as expected, at a low relative abundance, in accordance with its obligated intracellular localization in the upper genital tract, and not in the vaginal environment.

Other interesting data emerged from the investigation of the vaginal metabolic features in VVC-positive women, in particular the increased concentrations of vaginal glucose. High levels of glucose not only enhance the nutritive substrate of *Candida*, but also promote the expression of binding molecules in vaginal epithelial cells, thus increasing the adhesion of *Candida*^35,36^.

Notably, the higher levels of glucose found in VVC-positive women could by associated with the reduction of *L. crispatus* relative abundance in the vaginal environment. Indeed, it has been demonstrated that *L. crispatus* species shows a high metabolic activity in term of glucose consumption^37^.

During the switch from a commensal to a vaginal pathogen, *Candida* spp. produce various extracellular released enzymes, such as proteinases (Saps), phospholipases and hemolysins, that are implicated in adhesion, invasion and destruction of vaginal epithelial cells^38^. Some of the metabolic variations found in the vaginal ecosystem in VVC-positive women (e.g. higher levels of amino acids) could be ascribed to the tissue damage due to hydrolytic enzymes secreted by *Candida*.

In conclusion, in this paper, via 16S rRNA-based sequencing and ^1^H-NMR spectroscopy, we obtained deeper insights into the vaginal ecosystem of common infections of the female genital tract, such as VVC and *C. trachomatis* infection, on both a taxonomic and metabolomics point of view. We found that women suffering from VVC and CT conditions were characterized by a vaginal microbiome positioning between eubiosis (healthy women) and dysbiosis (BV-positive subjects), with a depletion of lactobacilli and a corresponding increase in different anaerobe genera (e.g. *Gardnerella*, *Prevotella*, *Megasphaera*, *Roseburia*, *Faecalibacterium* and *Atopobium*). The changes in the bacterial communities occurring during the genital infections correlated to significant alterations in the composition of vaginal metabolome, being the decrease of lactate concentration a common marker of all these infectious conditions.

From a clinical point of view, it has been already shown that several metabolites are significantly associated to BV clinical signs and symptoms, considering that this condition has strong metabolic signatures across multiple metabolic pathways^39^. Contrariwise, in VVC and CT infections, the quest for specific vaginal signatures (both taxonomic and metabolic) useful for clinical, diagnostic, or prognostic purposes is still an open issue. Our work sheds light on the less explored ecology of VVC and CT conditions, even if further investigations are needed to understand whether the microbial/metabolic alterations we identified precede the infection onset or if the pathogens themselves perturb the vaginal environment.

## Methods

### Study group and sample collection

Subjects included in this study are part of an enrolment occurred from January to July 2016 at the STI Outpatients Clinic of Sant’Orsola-Malpighi Hospital in Bologna (Italy). Samples collected during the enrolment were, partly, used for a first descriptive study of metabolome features in women affected by *C. trachomatis* infection. As previously described^20^, pre-menopausal heterosexual non-pregnant Caucasian women meeting the enrolment criteria (presence of urogenital symptoms or presence of risk factors for *C. trachomatis* infection) were included in the study and, then, subdivided in three groups on the basis of clinical and microbiological data. In particular, eligible women were allocated in one of the following groups: ‘Healthy’ (HC) (absence of symptoms and negative microbiological tests), ‘Bacterial Vaginosis’ (BV) (positivity for at least 3 out of 4 Amsel criteria, together with a Nugent score >3) and ‘Chlamydia’ (CT) (detection of *C. trachomatis* DNA by Versant CT/GC DNA 1.0 Assay; Siemens Healthineers, Tarrytown, NY, USA)^40^. In addition, a group of women suffering from vulvo-vaginal candidiasis (VVC) was included. VVC diagnosis was based on clinical symptoms (e.g. itching or vaginal whitish discharge), together with the microscopic and culture-based identification of *Candida*^41^. Samples were seeded on CHROMagar Candida Medium plates (Becton Dickinson, Franklin Lakes, NJ, USA) and incubated at 35 °C in aerobic atmosphere. The species identification of the grown yeast colonies was obtained by means of matrix-assisted laser desorption/ionization time-of-flight mass spectrometry (MALDI-TOF MS), using a Bruker Microflex MALDI-TOF MS instrument (Bruker Daltonics, Bremen, Germany). The presence of *Neisseria gonorrhoeae*, *Trichomonas vaginalis* and *Mycoplasma genitalium* was excluded by using the following molecular techniques: Versant CT/GC DNA 1.0 Assay (Siemens Healthineers), Aptima *T. vaginalis* and Aptima *M. genitalium* assays, Panther system (Hologic, Marlborough, MA, USA), respectively.

For all patients, demographic data and information about urogenital symptoms were recorded (Table 1) and two vaginal swabs were collected. The first one, collected by using E-swab collection system (Copan, Brescia, Italy) was used for microbiological diagnostic tests and BV assessment^20^. The second vaginal swab, collected with a sterile cotton bud and re-suspended in 1 ml of sterile saline, was centrifuged at 10,000 × g for 15 min. The obtained cell pellets were used for microbiome analysis, whereas the supernatants were employed for metabolome analysis, by means of ^1^H-NMR spectroscopy.

A written informed consent was obtained from all patients and the study protocol was approved by the Ethics Committee of Sant’Orsola-Malpighi Hospital, Bologna (7/2016/U/Tess). This study was carried out in accordance with the Declaration of Helsinki, following the recommendations of the Ethics Committee of the hospital.

### *Chlamydia trachomatis* genotyping

In case of a CT positive result, the correspondent remaining eluate was recovered from PCR plate and used for CT molecular genotyping^42^ by *omp1* gene semi-nested PCR followed by restriction fragment length polymorphism (RFLP) analysis, as previously described^40^. *C. trachomatis* serovar identification was achieved by the analysis of the specific restriction pattern.

### DNA extraction and library preparation

Genomic DNA was isolated from vaginal cell pellets using a DNeasy Blood and Tissue Kit (Qiagen, Hilden, Germany). DNA concentration and quality were determined using a NanoDrop ND‐1000 spectrophotometer (NanoDrop Technologies, Wilmington, DE, USA) and a TapeStation 2200 (Agilent Technologies, Santa Clara, CA, USA). The V3‐V4 hypervariable regions of the 16S ribosomal RNA (rRNA) gene were amplified according to the 16S Metagenomic Sequencing Library Preparation protocol (Illumina, San Diego, CA, USA) and sequenced on a MiSeq platform (Illumina), in a single 2 × 300 bp paired end run.

### Vaginal microbiota profiling

16S rRNA gene sequences were analyzed using PANDAseq (v. 2.5.0)^43^ and discarding low quality reads (i.e.: showing stretches of bases with a Q-score < 3 for more than 25% of their length); then, reads were processed using the QIIME pipeline (release 1.8.0)^44^ and clustered into Operational Taxonomic Units (OTUs) at 97% identity level. Taxonomic assignment was performed using RDP classifier^45^ against the Greengenes database (release 13.8; ftp://greengenes.microbio.me/greengenes_release/gg_13_8_otus), with a 0.5 identity threshold.

Species-level classification for all genera except *Lactobacillus* was performed by using SPINGO^46^ with default parameters. Characterization of *Lactobacillus* spp., on the other hand, was performed by BLAST-aligning all reads belonging to that genus to a custom reference database made up collecting all available reference sequences in NIH-NCBI database (ftp://ftp.ncbi.nlm.nih.gov/genomes/refseq/bacteria/) of 17 *Lactobacillus* species commonly found in the vaginal environment. Potential matches were filtered in order to retrieve an unequivocal classification for each read.

Co-abundance network analysis was performed as previously described^47^, using Spearman’s correlation between taxa and building hierarchical clusters of co-abundant groups (CAGs) at genus level by Spearman’s correlation metric and Ward linkage. Cytoscape (v 3.0)^48^ was used to graphically represent CAGs, as well as relative abundance of bacterial genera and strength of correlation. Additional details on species-level classification and CAG analysis can be found in Supplementary Methods.

Biodiversity and distribution of the microorganisms were characterized via alpha- and beta-diversity evaluations. Alpha-diversity was computed using Chao1, observed species, Shannon diversity and Faith’s Phylogenetic diversity (PD_whole_tree) metrics. Weighted and unweighted UniFrac distances were used for beta-diversity principal coordinates analysis (PCoA).

### Metabolomic analysis

Metabolomic analysis was performed starting from 700 µl of the cell-free supernatants of the vaginal swabs, added to 100 μl of a D_2_O solution of 3-(trimethylsilyl)-propionic-2,2,3,3-d4 acid sodium salt (TSP) 10 mM set to pH 7.0 by means of a 1 M phosphate buffer. ^1^H-NMR spectra were recorded at 298 K with an AVANCE III spectrometer (Bruker, Milan, Italy) operating at a frequency of 600.13 MHz, equipped with Topspin software (Ver. 3.5)^49^. The signals originating from large molecules were suppressed by a CPMG filter of 400 echoes, generated by 180° pulses of 24 μs separated by 400 μs^50^. The signals were assigned by comparing their multiplicity and chemical shift with Chenomx software data bank (ver 8.3, Chenomx Inc., Edmonton, Alberta, Canada). Separation among experimental groups on the basis of the metabolite profile was assessed via PCoA and “anosim” function on Bray Curtis and Euclidean distances between samples.

### Statistical analysis

Statistical evaluation among alpha-diversity indices was performed by a non-parametric Monte Carlo-based test, using 9999 random permutations in QIIME suite. “adonis” function of the R package “vegan” (v. 2.0–10, https://cran.r-project.org/web/packages/vegan/index.html) with 9999 random permutations was employed to determine statistical separation of the microbiota profiles. Differences in abundances of bacterial taxa and metabolites among experimental groups were analyzed by Kruskal-Wallis, followed by Dunn’s *post-hoc* tests. Metabolite concentrations were correlated to bacterial composition by calculating Spearman’s correlation coefficient between metabolites and bacterial genera present ≥1% in at least 1 sample. Strength of association between metabolites and bacterial profiles were assessed by both RV coefficient^51^ and Procrustes analysis. For each statistical analysis, unless otherwise stated, p-values < 0.05 were considered as significant. Statistical analyses were performed using MATLAB software (Natick, MA, USA).

## Supplementary information

Supplementary Information

## Data Availability

Raw reads are available in NCBI Short Read Archive (SRA, http://www.ncbi.nlm.nih.gov/sra) under accession number PRJNA523312.
