# Supplementary material for: Diversity of vaginal microbiome and metabolome during genital infections
Source: Sci Rep. 2019 Oct 1;9:14095. doi: 10.1038/s41598-019-50410-x (PMC6773718; doi:10.1038/s41598-019-50410-x)

Supplementary Information

Diversity of vaginal microbiome and metabolome during genital infections

Camilla Ceccarani[1,2^], Claudio Foschi[3^], Carola Parolin[4+], Antonietta D'Antuono[5], Valeria Gaspari[5], Clarissa Consolandi[1], Luca Laghi[6], Tania Camboni[1], Beatrice Vitali[4], Marco Severgnini[1*], Antonella Marangoni[3*]

[1] Institute of Biomedical Technologies, National Research Council, Segrate, Milan, Italy.
[2] Department of Health Sciences, San Paolo Hospital Medical School, University of Milan, Milan, Italy.
[3] Microbiology, Experimental Diagnostic and Specialty Department (DIMES), University of Bologna, Bologna, Italy.
[4] Department of Pharmacy and Biotechnology (FaBiT), University of Bologna, Bologna, Italy.
[5] Dermatology, St. Orsola-Malpighi Hospital, Bologna, Italy
[6] Centre of Foodomics, Department of Agricultural and Food Sciences, University of Bologna, Bologna, Italy

[^] Equal contribution to the work
[*] Jointly supervision of the work

[+] Corresponding author: Carola Parolin (carola.parolin@unibo.it)

SUPPLEMENTARY METHODS **3**

SUPPLEMENTARY REFERENCES 5

SUPPLEMENTARY FIGURES **7**

SUPPLEMENTARY FIGURE 1 8

SUPPLEMENTARY FIGURE 2 9

Supplementary Methods

Species-level analysis for *Lactobacillus* genus

Classification of reads belonging to *Lactobacillus*, the most important bacterial genus colonizing the vaginal enrironment, was further improved, where possible, down to the species level, via a BLAST-based [**S1**] re-classification on an *ad-hoc* built reference database. Due to the high similarity among *Lactobacillus* species in V3-V4 region of 16S rRNA, especially between *L. crispatus* (a well-known member of vaginal flora) and *L. gallinarum* ([**S2**], isolated in chicken crop and feces), we did not consider the whole set of *Lactobacillus* references available from NCBI RefSeq database for bacteria (ftp://ftp.ncbi.nlm.nih.gov/genomes/refseq/bacteria/), which comprised, as of 2018, a total of 518 strains.

Reference sequences

Genome sequences used for the classification comprised a total of 17 species among the most frequently found within the vaginal environment, for a total of 282 strains.

Through a custom script, sequenced genomes for all species and strains were downloaded and properly formatted for further processing. In all our analyses, only bacterial strains with a genome finishing grade of “Complete”, “Chromosome” or “Scaffolds” were considered. The following table summarizes the references used for each species.

**Species name**	**Number of strains**
Lactobacillus acidophilus	11
Lactobacillus brevis	11
Lactobacillus casei	18
Lactobacillus crispatus	15
Lactobacillus delbrueckii	28
Lactobacillus fermentum	15
Lactobacillus gasseri	10
Lactobacillus helveticus	15
Lactobacillus iners	2
Lactobacillus jensenii	13
Lactobacillus johnsonii	6
Lactobacillus paracasei	14
Lactobacillus plantarum	65
Lactobacillus reuteri	18
Lactobacillus rhamnosus	29
Lactobacillus salivarius	10
Lactobacillus vaginalis	2

Reads to re-classify

From the OTU table comprising all the samples, OTUs classified within the *Lactobacillus* genus were selected and the sequences of all the reads grouped in each OTU (clustered at 97% similarity) were retrieved. In order to reduce the number of sequences to re-classify, clonal reads (i.e.: reads being identical throughout 100% of their length and composition) were grouped together.

Classification

Re-classification of the reads was performed through nucleotide BLAST (legacy BLAST, v 2.26), using a cutoff of 1e-10 for the e-value and de-activating the dust-filter. Only reads matching for at least of 80% of their length were retained and, for each read, the best match (i.e.: that or those with the higher bit-score) was selected. If a read had multiple classifications on different species, the classification was reset to genus level.

Statistical analysis

In order to keep only consistent data for species-level evaluations, only samples having a relative abundance of *Lactobacillus* genus higher than 1%, were considered. This was made to exclude samples with very few reads classified in the genus that could profoundly alter the dataset (e.g.: considering a sample in which we had only 1 read in a genus, this would have brought a 100% to the species-level classification for that certain species). Since the least sequenced sample had about 12000 reads, this equaled having at least 120 reads in Lactobacillus genus; 75 out of the 79 samples were, thus, considered. As expected, all the 4 excluded samples were from women affected by bacterial vaginosis (BV), which is characterized by a dramatic decrease of *Lactobacillus* species and a consequent proliferation of other micro-organisms. A non-parametric Mann-Whitney U-test was used to compare the relative abundance of each bacterial species in the different experimental groups, considering p-values <0.05 as significant. Statistical evaluations were carried out using Matlab (v 2008b, Natick, MA, USA)

Co-abundance network analysis

Bacterial genera were selected considering only those present at >0.5% of abundance in at least 30% of the samples in at least one experimental group, in order to exclude minor and transient contributors of the gut microbiota. This resulted in a subset of 24 genera using the whole dataset, having a relative across all samples ranging from 55.80% to 0.17%.

All statistical evaluations and heatmaps were carried out using Matlab and the Fathom Toolbox [**S3**] and visualized by Cytoscape (v 3.0, [**S4**])

The co-abundance between each pair of genera was evaluated calculating the Spearman’s correlation coefficient and displayed as heatmaps, hierarchically clustered using Euclidean correlation metric and average linkage. Only associations having a Benjamini-Hochberg adjusted p-value <0.05 for the linear model were used to build for the hierarchical clustering. Results obtained considering the entire dataset of samples were used to define the co-abundance groups (CAGs). Permutational multivariate analysis of variance (P-MANOVA, [**S5**]) was used to determine whether CAGs were significantly different from each other. Essentially this compared strength of the correlations between the groups to correlation strengths within the groups in a pairwise manner. All comparisons, performed via 9999 random permutations, except for comparison between CAGs 2 *vs.* 3 and 2 *vs.* 4, had a p-value<0.05 for rejecting the hyphothesis of no-difference among groups. The whole PERMANOVA analysis resulted highly significant (p<0.001). Co-abundance network plots were created as previously described ([**S6**]). In the plots, circle and label size is proportional to the genus' relative abundance in the experimental group or along the whole dataset; circle colors represent the CAGs clusters; red edges suggest a positive correlation between genera, whereas blue edges represent negative correlation; edge thickness is proportional to the Spearman’s correlation coefficient.

Supplementary References

[S1] Altschul SF, Gish W, Miller W, Myers EW, Lipman DJ. Basic local alignment search tool. J Mol

Biol. 1990 Oct 5;215(3):403-10. PubMed PMID: 2231712

[S2] Fujisawa T, Benno Y, Yaeshima T, Mitsuoka T. Taxonomic study of the Lactobacillus acidophilus group, with recognition of Lactobacillus gallinarum sp. nov. and Lactobacillus johnsonii sp. nov. and synonymy of Lactobacillus acidophilus group A3 (Johnson et al. 1980) with the type strain of Lactobacillus amylovorus (Nakamura 1981). Int J Syst Bacteriol. 1992 Jul;42(3):487-91. PubMed PMID: 1503977.

[S3] Jones, D. L. 2015. Fathom Toolbox for Matlab: software for multivariate ecological and oceanographic data analysis. College of Marine Science, University of South Florida, St. Petersburg, FL, USA. Available from: http://www.marine.usf.edu/user/djones/matlab/matlab.html

[S4] Shannon P, Markiel A, Ozier O, Baliga NS, Wang JT, Ramage D, Amin N, Schwikowski B, Ideker T. Cytoscape: a software environment for integrated models of biomolecular interaction networks. Genome Res. 2003 Nov;13(11):2498-504. PubMed PMID: 14597658; PubMed Central PMCID: PMC403769.

[S5] Anderson, M.J. 2001. A new method for non-parametric multivariate analysis of variance. Austral Ecology, 26: 32–46.

[S6] Claesson MJ, Jeffery IB, Conde S, Power SE, O'Connor EM, Cusack S, Harris HM, Coakley M, Lakshminarayanan B, O'Sullivan O, Fitzgerald GF, Deane J, O'Connor M, Harnedy N, O'Connor K, O'Mahony D, van Sinderen D, Wallace M, Brennan L, Stanton C, Marchesi JR, Fitzgerald AP, Shanahan F, Hill C, Ross RP, O'Toole PW. Gut microbiota composition correlates with diet and health in the elderly. Nature.2012 Aug 9;488(7410):178-84. doi: 10.1038/nature11319. PubMed PMID: 22797518.

Supplementary Figures

Supplementary Figure 1. Definition of bacterial Co-abundance groups (CAGs). (a) Heatmap used to define CAGs, showing the Spearman correlation coefficient between genera and hierarchically clustered on the basis of Euclidean distance and Ward linkage. Only genera present at least at 0.5% relative abundance in at least 30% of the samples per experimental condition (i.e.: HC, VVC, CT, BV) are shown. Clustering is performed only on genera whose correlation is statistically different from 0 (p-value of the linear model <0.05). **(b)** Network plot highlighting correlation relationships of CAGs for the whole cohort studied (n=79). Circle sizes indicate genus abundances and line thickness is proportional to correlation value. Red lines indicate a positive correlation value; blue lines a negative one. **(c)** Bar plots showing the average cumulative relative abundance of each CAG in the microbiota of the subjects for each experimental group. In grey, the portion of genera not belonging to the identified CAGs due to the initial filtering is represented.

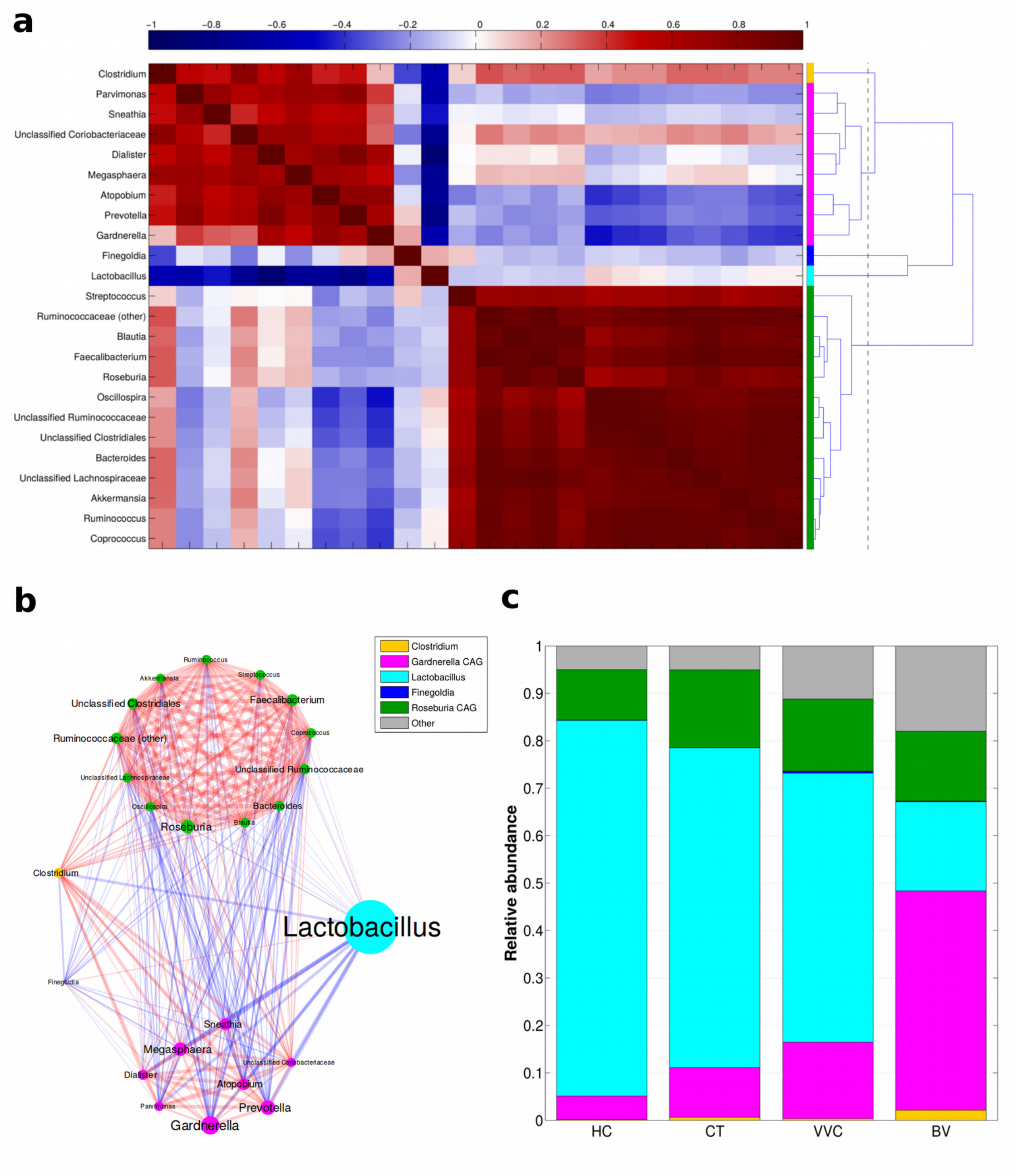

Supplementary Figure 2. **Co-abundance groups (CAG) networks**. Taxonomic correlations among CAGs in **(a)** healthy (HC), **(b)** *C. trachomatis* (CT), **(c)** vulvo-vaginal candidiasis (VVC) and **(d)** bacterial vaginosis (BV) positive women. Red edges indicate a positive correlation, while blue edges a negative one. Edge size is proportional to the correlation coefficient. Node and label size represent taxonomy abundance, while the color indicates the belonging cluster: *Lactobacillus* in cyan, *Roseburia* CAG in green, *Gardnerella* CAG in magenta, *Clostridium* in yellow, and *Finegoldia* in blue.

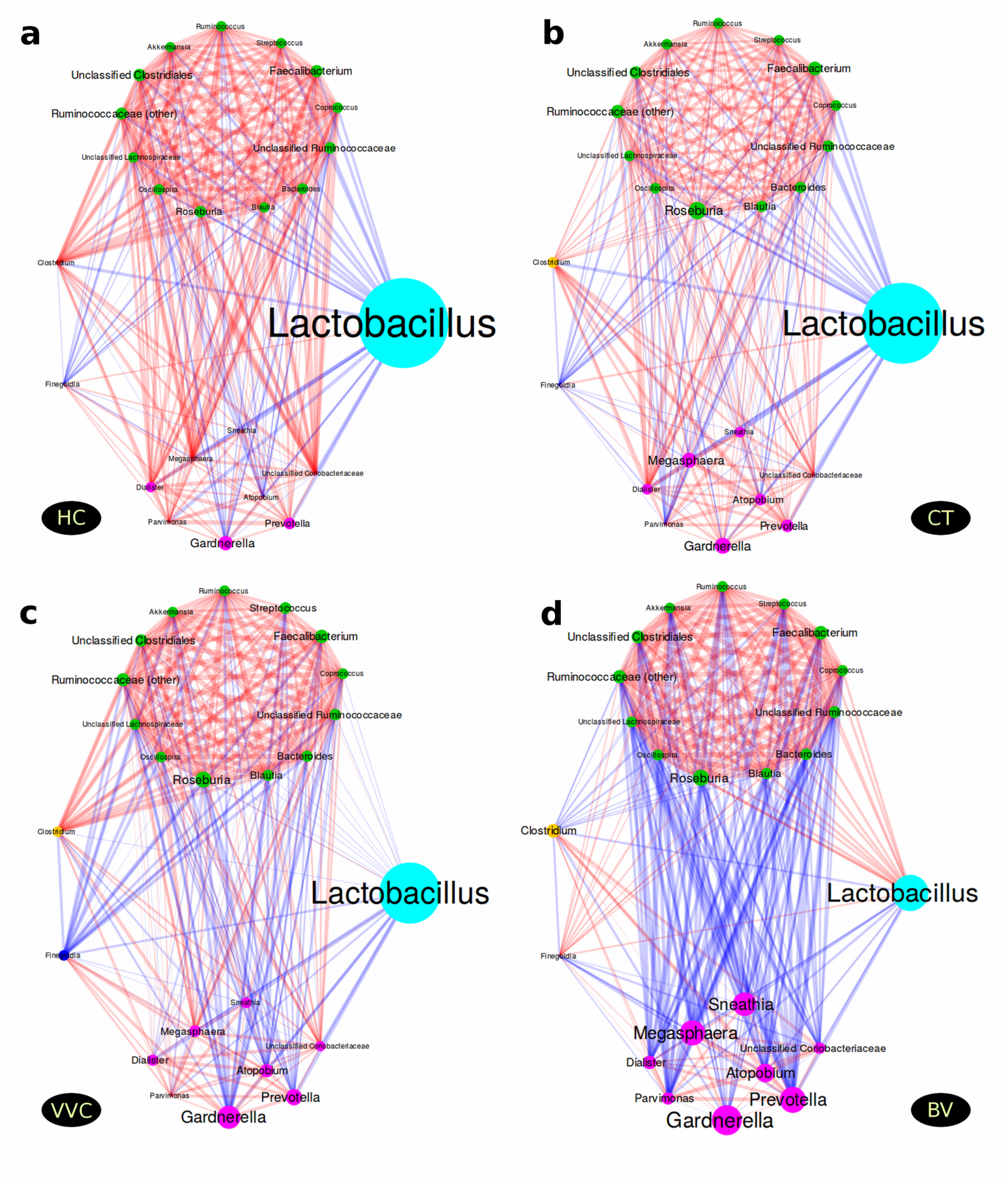

Supplement: Supplementary file 1 — Supplementary Information [file 41598_2019_50410_MOESM1_ESM.pdf]
